# COVID-19 and tinnitus—a tertiary care Centre experience

**DOI:** 10.1186/s43163-022-00255-5

**Published:** 2022-05-21

**Authors:** Aditiya Saraf, Monika Manhas, Parmod Kalsotra, Raies Ahmad

**Affiliations:** 1 Department of ENT and Head and Neck Surgery, SMGS Hospital, Government Medical College Jammu, Jammu, Jammu and Kashmir, India; 2 Department of Physiology, Government Medical College Jammu, Jammu, Jammu and Kashmir, India

**Keywords:** COVID-19, Tinnitus, Inventory, Psychological

## Abstract

**Background:**

COVID-19 outbreak was declared a global pandemic in March 2020 by WHO. Due to person-to-person transmission of this infection, majority of countries of world introduced lockdown to ensure people stay at home. A complex bidirectional interaction exists between tinnitus and emotional distress, as they can exacerbate one another. Also, COVID-19 infection can cause damage to outer hair cells. The aim of this study is to find out relationship between COVID-19 and initiation or exacerbation of tinnitus.

**Methods:**

The present retrospective study, after approval by Institutional Ethics Committee, was conducted in Department of ENT, GMC Jammu, from June 2020 to March 2021 on 90 patients with primary complaint of tinnitus. All 90 patients were asked to complete the Tinnitus Handicap Inventory. Also, all patients were asked COVID-19-related questions (history of contracting virus/history of grief/anxiety/depression/stress/nervousness/financial status/ physical exercise/sleep routine/ social interactions).

**Results:**

Out of 90 patients, 72 patients (80%) had chronic longstanding tinnitus and 18 patients (20%) experienced tinnitus during pandemic. Out of 72 patients who gave history of longstanding tinnitus, 41 patients (56.9%) reported tinnitus to be stable during COVID-19 pandemic, 26 patients (36.1%) reported it to become more bothersome and 5 patients (6.9%) reported that tinnitus was improved. Out of 90 patients, 21 patients (23.3%) were diagnosed as COVID-19 positive. Of these 21 patients experiencing COVID-19 symptoms, 16 patients (76.2%) gave history of exacerbation of tinnitus, 4 patients (19%) gave history of tinnitus remaining stable and 1 patient (4.7%) said that tinnitus was improved. Out of 90 patients, history of negative psychological impact due to COVID-19 restrictions was seen in 65 patients (72.2%).

**Conclusion:**

With our study, we concluded that ENT specialists should be fully aware that not only pre-existing tinnitus may be exaggerated due to COVID-19 infection, but also, there can be development of new-onset tinnitus due to COVID-19 infection and the negative psychological impact due to COVID-19 pandemic.

## Background

COVID-19 or coronavirus disease 2019 is an infectious respiratory disease caused by severe acute respiratory syndrome coronavirus 2. COVID-19 outbreak was declared a global pandemic in March 2020 by WHO [14].

Due to person-to-person transmission of this infection, majority of countries of the world introduced lockdown to ensure people stay at home and also advised social distancing, in order to prevent spread of infection. However, all these measures, although, efficiently curbed the spread of this deadly virus—the fear of contracting disease, poor sleep quality, financial burden and social isolation led to depression and anxiety in people [6].

The term ‘tinnitus’ comes from a Latin word *tinnire* meaning ‘to ring’ and was introduced by Pliny. Tinnitus is an auditory perception due to altered spontaneous activity within auditory pathway, arising as a result of aberrant excitation or inhibition. The underlying mechanisms for tinnitus can be abnormal afferent excitation at cochlear level (glutamate neuro-excito-toxicity, modulation of NMDA and non-NMDA receptors, calcium channel dysfunction), efferent dysfunction (reduction of GABA effect) and alteration of spontaneous activity and tonotopic reorganisation [4].

Tinnitus can be classified as subjective or objective. Subjective tinnitus means origin may be within the external ear, middle ear, inner ear, VIII nerve, or central nervous system. Objective tinnitus may be seen in vascular conditions (like AV shunts, carotid aneurysm, persistent stapedial artery, dehiscent jugular bulb), patulous Eustachian tube, temporomandibular joint abnormality, etc. [4].

A complex bidirectional interaction exists between tinnitus and emotional distress, as they can exacerbate one another. Due to COVID-19 pandemic, there has been increase in negative psychological effect on people leading to stress. Tinnitus commonly spikes or is initiated during stress because of two related systems—(1) brain regions along hypothalamic-pituitary-adrenal axis, which is main area involved in stress response, and (2) limbic system (hippocampus and amygdala) which regulates tinnitus perception and adaptation, and ability to cope stress. In addition, due to widespread lockdowns, it became very difficult to seek medical advice for non-life-threatening conditions like tinnitus [7].

Also, COVID-19 infection can cause damage to outer hair cells, as has been evidenced by the reduced amplitude of transient evoked otoacoustic emissions (TEOAEs) and distortion product otoacoustic emissions (DPOAEs) [5].

At present, there are very few published research articles showing relationship between COVID-19 and tinnitus, especially in this local population. With our study, we aim to find out relationship between COVID-19 and initiation or exacerbation of tinnitus.

## Methods

The present retrospective study, after approval by Institutional Ethics Committee, was conducted in the Department of ENT, GMC Jammu, from June 2020 to March 2021 on 90 patients with primary complaint of tinnitus.

Inclusion criteria are as follows: age above 20 years, absence of metabolic disorders like thyroid disorders and hyperlipidemia and absence of cardiovascular diseases like hypertension, anaemia and arrhythmias.

Exclusion criteria are as follows: history of middle ear disease, history of ear surgery, pregnancy and history of head injury/brain haemorrhage/brain infarct.

All 90 patients were asked relevant clinical history. All patients were subjected to general physical and systemic examination. Local ENT examination including otoscopy and tuning fork tests was done on all patients. Pure tone audiometry was performed on all patients.

Informed consent was taken and all patients were asked to complete the THI-Tinnitus Handicap Inventory [9] to know the severity of tinnitus. THI is a validated measure of tinnitus handicap and comprises of 25 questions. It is scored from 0 (slight handicap) to 100 (catastrophic handicap).

All patients were also asked COVID-19-related questions regarding stress due to social isolation/distancing, history of contracting COVID-19 infection and medication thereof, any financial uncertainty due to lockdown and information related to social interactions/ physical exercises/ sleep routine during lockdown.

All data was entered in Microsoft Excel SpreadSheet and analysed using the Statistical Package for Social Sciences (SPSS) software (version 21 for windows). Appropriate statistical analytical tests were applied were applied as per the advice of statistician.

## Results

The mean age of presentation was 39.06 ± 7.66 years, with maximum number of patients in the age group of 35–50 years (Table 1)—younger population, this finding being statistically insignificant (*p* value= 0.65). Out of 90 patients, 58 were females (64.4%) and 32 were males (35.5%), indicating statistically significant female preponderance (*p* <0.05).

**Table 1:** Age distribution

Age group (in years)	Number of patients (*n*=90)
20 to less than 35	12 (13.3%)
35 to less than 50	38 (42.2%)
50 to less than 65	29 (32.2%)
More than 65	11 (12.2%)

**Fig. 1:**
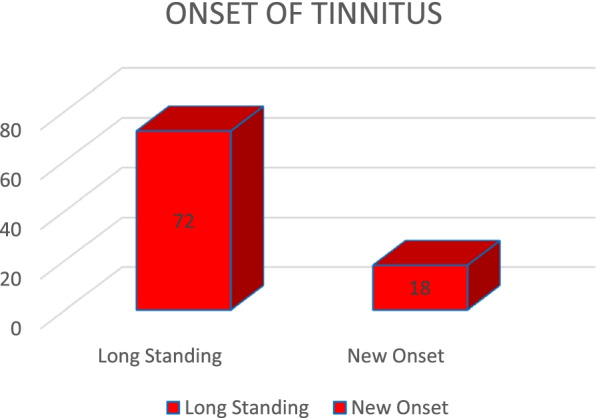
Distribution of patients according to onset of tinnitus

The mean duration of tinnitus was 2.22 ± 0.83 years. Out of 90 patients, 72 patients (80%) had chronic longstanding tinnitus and 18 patients (20%) experienced tinnitus during pandemic (Fig. 1), this finding being statistically significant (*p*<0.05). Tinnitus was bilateral in 31 patients (34.4%) and unilateral in 59 patients (65.5%), the observation being statistically significant (*p* value<0.05).

The mean THI score was 43.6—out of 90 patients coming to ENT OPD with tinnitus (Table 2), 17 patients had slight handicap (18.8%), 33 patients had mild handicap (36.6%), 38 patients had moderate handicap (42.2%) and 2 patients had severe handicap (2.2%), these observations being statistically insignificant (*p* value=0.68).

**Table 2:** Distribution of patients according to Tinnitus Handicap Inventory

Handicap on THI	Number of patients (*n*=90)
Slight	17 (18.8%)
Mild	33 (36.6%)
Moderate	38 (42.2%)
Severe	2 (2.2%)
Catastrophic	Nil

**Fig. 2:**
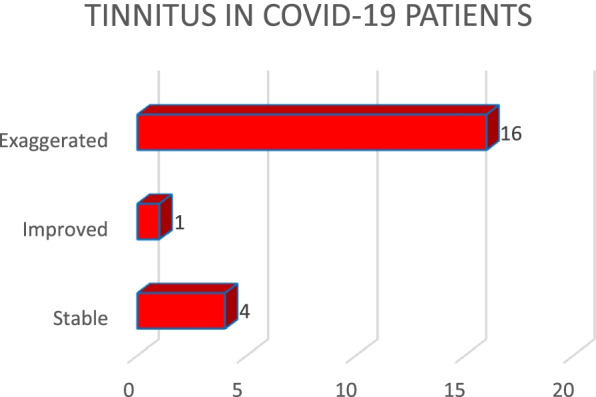
Experience of tinnitus among COVID-19 patients

Out of 72 patients who gave history of longstanding tinnitus, 41 patients (56.9%) reported tinnitus to be stable during COVID-19 pandemic, 26 patients (36.1%) reported it to become more bothersome and 5 patients (6.9%) reported that tinnitus was improved, this finding being statistically significant (*p* value <0.05). Out of 90 patients, 21 patients (23.3%) were diagnosed as COVID-19 positive. Of these 21 patients experiencing COVID-19 symptoms, 16 patients (76.2%) gave history of exacerbation of tinnitus, 4 patients (19%) gave history of tinnitus remaining stable and 1 patient (4.7%) said that tinnitus was improved (Fig. 2).

Out of the 21 COVID-19-positive patients, 14 patients (66.6%) had received hydroxychloroquine (ototoxic drug), while other 7 patients (33.3%) did not take hydroxychloroquine, this finding being statistically insignificant (*p* value =0.54).

**Fig. 3:**
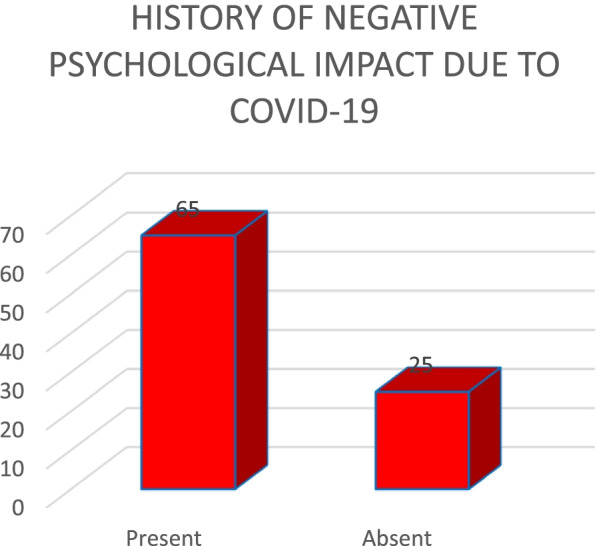
History of negative psychological impact due to COVID-19

Out of 90 patients (Fig. 3), history of negative psychological impact (grief/frustration/nervousness/stress/anxiety/depression) due to COVID-19 restrictions was seen in 65 patients (72.2%), the finding being statistically significant (*p* value <0.05).

## Discussion

Tinnitus, a form of auditory dysfunction, is a common condition that is recognised as a major health issue by about 0.5% of world’s population. Tinnitus is a form of sensory epilepsy in which brain generates an abnormal memory of a sensation which is sustained long after the sensory receptors have been damaged, that appears to be associated with burst firing due to a decrease in GABAergic inhibition and increase in glutamatergic excitation [13].

Dynamics of emotion are linked with course of tinnitus, which adds to the complex interaction of tinnitus distress, tinnitus loudness, stress and emotional perception. Any factor that can alter the tinnitus coping strategies (like social and physical activation, etc.) leads to increase of tinnitus. The environmental and social factors during COVID-19 pandemic and associated government lockdown measures potentially limited all the coping strategies for tinnitus [11].

The mean age of presentation was 39.06 ± 7.66 years, with maximum number of patients in the age group of 35–50 years (Table 1). This observation was comparable to studies conducted by Chirakkal et al. and Xia et al. [5, 16]. Out of 90 patients, 58 were females (64.4%) and 32 were males (35.5%), this observation being consistent with findings of Beukes et al. [3]. More involvement of young and female group could be due to lifestyle changes in these groups during pandemic such as increased childcare, increased household responsibilities and changes in employment. However, Schlee et al. in their study concluded that there was more involvement of elderly and male population [12].

The mean duration of tinnitus was 2.22 ± 0.83 years. Out of 90 patients, 72 patients (80%) had chronic longstanding tinnitus and 18 patients (20%) experienced tinnitus during pandemic (Fig. 1). Tinnitus was bilateral in 31 patients (34.4%) and unilateral in 59 patients (65.5%). Our study showed that most patients had long standing tinnitus, which is comparable to studies conducted by Beukes et al. and Beukes and Manchaiah [2, 3].

The mean THI score was 43.6—out of 90 patients attending ENT OPD with tinnitus (Table 2), 17 patients had slight handicap (18.8%), 33 patients had mild handicap (36.6%), 38 patients had moderate handicap (42.2%) and 2 patients had severe handicap (2.2%), these observations being statistically insignificant (*p* value=0.68). Out of 72 patients who gave history of longstanding tinnitus, 41 patients (56.9%) reported tinnitus to be stable during COVID-19 pandemic, 26 patients (36.1%) reported it to become more bothersome and 5 patients (6.9%) reported that tinnitus was improved. These findings were consistent with study conducted by Xia et al. [16]. The possible reason that few patients reported their tinnitus remained stable could be due to many factors such as patient or patient’s family not contracting disease, acceptance of new routine, or no financial changes. In addition, minority of patients experienced tinnitus to improve; possible reason for it could be due to better sleep, doing more relaxation exercises, continuing social interactions, etc. [3].

Out of 90 patients, 21 patients (23.3%) were diagnosed as COVID-19 positive. Of these 21 patients experiencing COVID-19 symptoms, 16 patients (76.2%) gave history of exacerbation of tinnitus, 4 patients (19%) gave history of tinnitus remaining stable and 1 patient (4.7%) said that tinnitus was improved (Fig. 2). This observation was comparable to study conducted by Buekes et al. and Xia et al. [3, 16]. The possible reason for COVID-19 patients to experience bothersome tinnitus or pre-existing tinnitus being exaggerated might be due to the fact that COVID-19 infection has deleterious effects on the outer hair cells in the cochlea. The viral particles can damage the auditory system by direct damage to organ of Corti, ischaemic damage to stria vascularis or spiral ganglion. There have been some studies indicating viral damage to brainstem as well [5].

Out of the 21 COVID-19-positive patients, 14 patients (66.6%) had received hydroxychloroquine (ototoxic drug), while other 7 patients (33.3%) did not take hydroxychloroquine. In our study, 14 patients had received a potentially ototoxic drug, hydroxychloroquine, during COVID-19 treatment. This drug causes ototoxicity by damaging outer hair cells of cochlea and inhibition of post-synaptic sodium channel function in spiral ganglion cells. Also, COVID-19 patients not receiving hydroxychloroquine can still develop tinnitus, due to hypoxia which has deleterious effects on stria vascularis [10].

Out of 90 patients (Fig. 3), negative psychological impact (grief/frustration/stress/anxiety/depression) due to COVID-19 restrictions was seen in 65 patients (72.2%). This observation was consistent with studies conducted by Viola et al., Xia et al. and Beukes et al. [3, 15, 16]. The possible reason for this might be due to the fact that stress causes tinnitus to acquire a negative emotional significance through maladaptive cognatic appraisal and dysfunctional processes in the autonomic nervous system; as a result of which negative catastrophic interpretations of tinnitus perception are sustained and tinnitus habituation does not occur [1, 8].

## Conclusion

COVID-19 pandemic has disrupted every domain of our lives—the way people socialise, the way people interact and the way people assess medical facilities. Also, as this pandemic may remain for considerable time in future as well, the health, social and emotional implications are likely to continue.

With our study, we concluded that ENT specialists should be fully aware that tinnitus can be caused by contracting COVID-19 infection or pre-existing tinnitus may be exaggerated. Also, negative psychological impact due to COVID-19 has substantially increased the problem of tinnitus.

## Data Availability

Available with corresponding author upon reasonable request
